# Enhanced Wettability and Adhesive Property of PTFE through Surface Modification with Fluorinated Compounds

**DOI:** 10.3390/ma17133051

**Published:** 2024-06-21

**Authors:** Shakila Parveen Asrafali, Thirukumaran Periyasamy, Seong-Cheol Kim, Jae-Woong Lee

**Affiliations:** 1 Department of Fiber System Engineering, Yeungnam University, Gyeongsan 38541, Republic of Korea; shakilaparveen@yu.ac.kr (S.P.A.); thirukumaran@ynu.ac.kr (T.P.); 2 School of Chemical Engineering, Yeungnam University, Gyeongsan 38541, Republic of Korea; sckim07@ynu.ac.kr

**Keywords:** surface modification, Teflon, fluoro-compound, plasma treatment, hydrophilic surface

## Abstract

Polytetrafluoroethylene (PTFE) is prized for its unique properties in electrical applications, but its natural hydrophobicity poses challenges as it repels water and can cause electrical short circuits, shortening equipment lifespan. In this work, the mentioned issue has been tackled by using two different fluorinated compounds, such as perfluorooctanoic acid (PFOA)/perfluorooctanol (PFOL), along with plasma processing to enhance the surface hydrophilicity (water attraction) of PTFE. This method, demonstrated on Teflon membrane, quickly transformed their surfaces from hydrophobic to hydrophilic in less than 30 s. The treated films achieved a water contact angle saturation of around 80°, indicating a significant increase in water affinity. High-resolution C 1s X-ray photoelectron spectroscopy (XPS) confirmed the formation of new bonds, such as -COOH and -OH, on the surface, responsible for enhanced hydrophilicity. Extended plasma treatment led to further structural changes, evidenced by increased intensity in infrared (IR) and Raman spectra, particularly sensitive to vibrations associated with the C-F bond. Moreover, Attenuated Total Reflectance Fourier-Transform Infrared Spectroscopy (ATR-FTIR) showed the formation of surface-linked functional groups, which contributed to the improved water attraction. These findings decisively show that treatment with fluoro-compound along with plasma processing can be considered as a highly effective and rapid method for converting PTFE surfaces from hydrophobic to hydrophilic, facilitating its broader use in various electrical applications.

## 1. Introduction

The interaction between liquids and solid surfaces orchestrates a fascinating phenomenon: the creation of hydrophobic (water-repelling) and hydrophilic (water-attracting) surfaces. Two main factors govern this surface behavior: wettability, describing how a liquid spreads on a solid, and adhesion, determining the strength of the bond between them. Understanding and controlling these interactions are crucial because both hydrophobic and hydrophilic materials play vital roles in our daily lives. Superhydrophobic surfaces, inspired by the lotus effect, demonstrate exceptional water repellency. Water droplets bead up and roll off effortlessly, making these surfaces self-cleaning, anti-icing, and even anti-bacterial [1,2,3,4,5]. Conversely, superhydrophilic surfaces boast excellent water wettability, spreading water readily. These surfaces find applications in biocompatible materials and anti-fog coatings. Beyond these extremes lies the intriguing case of gecko surfaces, also known as adhesive hydrophobic surfaces. Despite their increased water contact angles indicating repellency, gecko surfaces can hold water droplets in place, making them ideal for precise microfluidic manipulation [6,7,8,9].

Polytetrafluoroethylene (PTFE), widely known as Teflon, serves as a prime example of a superhydrophobic material. Its fluorocarbon structure grants it a remarkable water contact angle exceeding 140°. This property makes PTFE a cost-effective choice for superhydrophobic layers in various electro-wetting on dielectric applications. Its versatility extends further into textiles, household items, sealing solutions, electronics, and even environmental protection. Notably, PTFE boasts exceptional resilience in harsh environments due to its high resistance to acids, alkalis, and corrosion. However, in electronic applications, the inherent porosity of PTFE membranes can become a liability, allowing electrical breakdown, leading to short circuits, and ultimately reducing the material’s lifespan [10,11,12,13]. Hence, the imperative to manage the surface characteristics of PTFE while safeguarding its inherent properties emerges as a pressing need, especially in the quest to prolong the lifespan of electronic components. Fortunately, a diverse arsenal of methodologies stands ready to accomplish this surface refinement. Among these, chemical approaches stand out, involving treatments employing hydrophilic agents through various application methods such as dip coating, spin coating, or spray coating. Conversely, physical techniques like plasma treatment and electron beam irradiation offer nuanced precision in effecting surface alterations [14,15,16,17]. It is essential to underscore that, irrespective of the chosen fabrication method, meticulous oversight over the morphology and dimensions of microscopic and nanoscopic entities on the surface remains paramount. These attributes serve as the linchpin for attaining the desired hydrophobic or hydrophilic behavior.

Plasma treatment has emerged as a game-changer in surface modification, offering a trifecta of benefits: affordability, environmental friendliness, and scalability. This technique allows for precise manipulation of a material’s surface characteristics without altering its bulk properties. It achieves this remarkable feat through a combination of physical and chemical processes. The magic lies in the interaction between the material’s surface and a highly reactive, ionized gas state known as plasma. This plasma bombards the surface with energetic particles, triggering a cascade of effects. Energetic ions physically etch the surface, modifying its topography. Simultaneously, chemical reactions occur between the plasma and the surface molecules, altering the surface chemistry. Additionally, plasma treatment can induce cross-linking, essentially creating a network of bonds between surface molecules, further enhancing certain properties. The beauty of plasma treatment lies in its controllability. By fine-tuning various operational parameters—pressure, power, treatment time, and gas flow rate—a wide range of surface functionalities can be achieved. Furthermore, the choice of plasma gas plays a critical role. Common gases used include argon (Ar), nitrogen (N_2_), oxygen (O_2_), hydrogen mixtures (Ar+H_2_), carbon dioxide (CO_2_), and ammonia (NH_3_). Each gas interacts differently with the surface, leading to diverse outcomes. This precise control is exemplified by the research of Liu et al. [2]. They investigated the effects of plasma treatment on three distinct polymer surfaces: polytetrafluoroethylene (PTFE), polyimide (PI), and poly (lactic acid) (PLA). Interestingly, they found that the optimal treatment parameters varied for each polymer. PTFE and PI exhibited a strong dependence on the duration of plasma exposure, while PLA’s response was most sensitive to the distance between the electrodes. Nevertheless, all three polymers showcased a significant improvement in wettability under various settings. This observation underscores the profound changes plasma treatment can induce in surface chemistry, with the ability to transform a hydrophobic surface (repels water) to hydrophilic (attracts water) and vice versa. Sprick et al. [4] enhanced the photocatalytic performance of conjugated polymer film bearing apolar sidechains, FS-5Dodec, which is a copolymer of a dibenzo [b, d] thiophene sulfone monomer and di-n-dodecyl-9H-fluorene, by enhancing its wettability through plasma treatment. They found that the plasma treatment duration of 20 min is enough to create a chemically modified surface possessing a reduced contact angle of 43° and an eight-fold increase in hydrogen evolution rate. Ji et al. [18] modified the surface of two fluorine resin membranes, ethylene tetrafluoroethylene (ETFE) and polytetrafluoroethylene (PTFE) membranes, by means of atmospheric pressure dielectric barrier discharge (DBD) plasma. The He/AA (helium/acrylic acid) plasma treatment results in producing stable grafted polyacrylic acid (g-PAA) on the membrane surface and improved the polarity and adhesion strength of the fluoro-resin membranes with decreased water contact angle values from 95.83° to 49.9° for ETFE membrane and from 109.9° to 67.8° for PTFE membrane. Additionally, the incorporation of specific functional groups or atoms onto the surface can further tailor its properties for specific applications. In essence, plasma treatment offers a powerful and versatile approach to surface modification. Its ability to precisely control surface properties while maintaining the bulk integrity of the material makes it a valuable tool across various industries [19,20,21,22,23,24].

The novelty of the work lies in adopting both physical (plasma treatment) and chemical treatment (dip coating) methods. In previous reports, the authors used plasma treatments alone or the hydrophilic compound is passed along with the active gas. Expanding on our previous research [23] on inducing hydrophilicity in PTFE through plasma treatment, this study goes further to enhance the stability of the modified surface. We previously examined various active gases and treatment durations and found that plasma treatment using ‘Ar’ as carrier gas and ‘N_2_’ as active gas for 2 min of plasma duration was most effective in reducing the water contact angle of PTFE from 135° to 78°. However, a notable limitation was the temporary nature of the achieved hydrophilicity. To overcome this hurdle, we propose a novel two-step approach. In this method, we utilize dip coating to apply two different fluoro-compounds—perfluorooctanoic acid and perfluorooctanol—onto the PTFE surface. The reason for choosing fluoro-compounds is that the hydrophobic fluoro part will stick to the membrane surface, and the acid and alcohol functional groups will protrude outside, creating hydrophilicity. Subsequently, plasma treatment is conducted on these coated surfaces. This combined strategy aims to achieve a more significant reduction in the contact angle while ensuring long-term stability in the wettability. The main application of this work is the property enhancement by creating stable hydrophilic PTFE surfaces. This work includes a thorough analysis of all pertinent material characterizations, offering a detailed insight into the achieved surface modifications.

## 2. Materials

The PTFE sheet roll (0.08 mm thick, 20 cm long) was bought from Flontec Co., Ltd. (Incheon, Republic of Korea). Argon and N_2_ gas were supplied by Korea Standard Gas (KSG), the Republic of Korea. Perfluorooctanoic acid and perfluorooctanol were purchased from Sigma Aldrich (St. Louis, MO, USA), while dichloromethane was obtained from Daesung Chemicals (Daegu, Republic of Korea). All chemicals and solvents were used without additional purification.

## 3. Methods

### 3.1. Preparation of Coating Solutions

Two different coating solutions were prepared using PFOA and PFOL. PFOA and PFOL solutions containing 10 wt.% were prepared using dichloromethane as a solvent.

### 3.2. Modification of Polytetrafluoroethylene (PTFE)

**Scheme 1:**
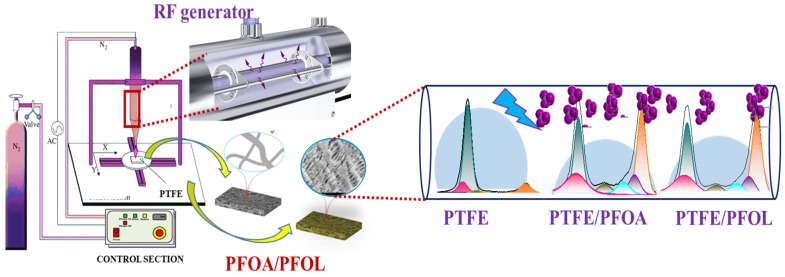
Schematic representation showing the surface modification process of PTFE.

Modification of PTFE was executed using two distinct processes: immersion in perfluorooctanoic acid (PFOA) and plasma treatment. To begin, a PTFE sheet measuring 10 × 10 cm was immersed in a 10% PFOA solution contained in a large petri dish. The sheet was carefully placed to ensure it was fully submerged without any folds, resulting in a fully wetted and transparent appearance. After 10 min of immersion, the PTFE sheet was removed and laid flat to dry in the open air. During the drying process, the transparent sheet gradually regained its original texture and color. Once the PTFE was completely dry, it was prepared for the second modification stage involving N_2_ plasma treatment. For the plasma treatment, the dried PTFE sheet was positioned on the sample holder of the plasma equipment, ensuring secure placement. Based on prior research [23], it was established that using nitrogen (N_2_) as the active gas with a 2-min plasma exposure effectively produced a hydrophilic surface. Accordingly, the procedure was replicated here, with specific flow rates set at 4.5 L/min for the carrier gas (argon) and 20 mL/min for the active gas (nitrogen), with an RF power of 150 W and maintaining the distance between the plasma nozzle and Teflon sheet to 3 mm. Three different plasma exposure durations—10, 20, and 30 s—were applied (as illustrated in Scheme 1). Immediately after plasma treatment, the modified PTFE was sealed in a zipper bag to prevent contact with the atmosphere. A similar protocol was followed for PTFE modification using perfluorooctyl acrylate (PFOL). All modified samples were meticulously packed and subsequently subjected to a series of characterizations to assess their properties.

### 3.3. Expanded Description of Characterization Techniques

#### 3.3.1. Fourier-Transform Infrared Spectroscopy (FTIR)

A PerkinElmer MB3000 ATR FTIR spectrometer (Waltham, MA, USA) was used to acquire infrared absorption spectra of both neat (untreated) and plasma-treated PTFE samples. The instrument analyzed a wide range of wavenumbers, from 400 to 4000 cm⁻^1^. To ensure data consistency, spectra were collected from three distinct locations on each PTFE sample and placed onto the sample disc.

#### 3.3.2. Raman Spectroscopy

Raman spectra were obtained using a Horiba XploRA Micro-Raman spectrophotometer located in Palaiseau, France. This technique probes the vibrational modes of molecules and provides complementary information to FTIR. The Raman spectra covered a wavenumber range of 500 to 4000 cm⁻^1^, offering insights into similar functional groups as FTIR but potentially with different selection rules.

#### 3.3.3. X-ray Photoelectron Spectroscopy (XPS)

High-resolution XPS measurements were performed using a Thermo Scientific K-Alpha instrument (Waltham, MA, USA, Ion source energy: 100 V to 3 keV, angle resolved in XPS: 0~60 degree) to investigate the elemental composition and chemical state of the PTFE surface and modified PTFE surface. The resulting data were deconvoluted using CasaXPS (2.3.22PR1.0) software to separate overlapping peaks and identify the specific chemical environments of the elements present.

#### 3.3.4. Electron Microscopy

The morphology (surface features) of PTFE and modified PTFE samples were characterized using two complementary electron microscopy techniques:

Field Emission Scanning Electron Microscopy (FESEM) with energy-dispersive X-ray spectroscopy (EDS): this instrument provided high-resolution images of the surface at various magnifications (1000× to 10,000×). Additionally, EDS provided elemental analysis at specific points on the sample surface. The FESEM operated with an accelerating voltage of 10 kV and a working distance of approximately 8–9 mm.

High-Resolution Transmission Electron Microscopy (HRTEM): this instrument offered atomic-scale resolution, allowing for detailed observation of the microstructure of the PTFE and its modified surface.

#### 3.3.5. Apparent Contact Angle Measurement

The wettability of PTFE and modified PTFE membranes was evaluated using a Dataphysics Instrument OCA 20 model contact angle goniometer (Stuttgart, Germany). The PTFE membranes were carefully placed horizontally on glass slides. Using a micro-syringe, a small and precise volume (around 2 microliters) of deionized (DI) water was dispensed onto each membrane. The contact angle, which reflects the interaction between the water droplet and the membrane surface, was measured at five different locations on each sample, and their average value was calculated, denoting the apparent contact angle. The instrument can measure contact angles ranging from 0° (perfectly wetting) to 180° (completely non-wetting).

## 4. Results and Discussion

### 4.1. FTIR Analysis

**Figure 1:**
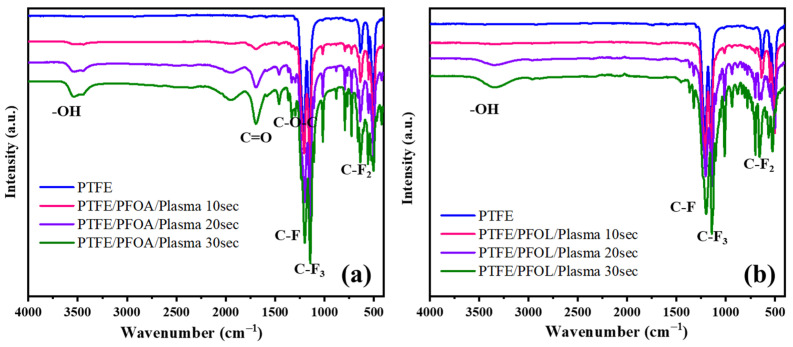
FTIR spectra of (**a**) PTFE and PTFE/PFOA/N_2_ plasma and (**b**) PTFE and PTFE/PFOL/N_2_ plasma.

In the process of treating PTFE with perfluorooctanoic acid and perfluorooctanol, these compounds bond to the PTFE’s surface. This is followed by nitrogen (N_2_) plasma treatment, which removes fluorine atoms and creates carbon–nitrogen (C-N) bonds. These C-N bonds make the surface more water-attracting (hydrophilic). The chemical changes in PTFE. after treatment with PFOA or PFOL followed by N_2_ plasma, were confirmed using Fourier-Transform Infrared Spectroscopy (FTIR). Figure 1a shows the FTIR spectra of PTFE and PTFE treated with PFOA/N_2_ plasma. The untreated PTFE spectrum shows peaks at 1205 and 1149 cm^−1^ corresponding to vibrations of carbon–fluorine (C-F) bonds and additional peaks at 638, 525, and 501 cm^−1^ due to bending vibrations of the carbon–fluorine chains. The treated PTFE shows new peaks compared to the untreated material, indicating the presence of the fluorinated compounds and the effects of the plasma treatment. PFOA specifically creates new peaks at 3600 cm^−1^ (due to the O-H group), 1720 cm^−1^ (due to the C=O group), and 1250 cm^−1^ (due to C-O-C stretching). There is also a broad, weak peak around 1600 cm^−1^, which indicates water molecules adsorbed on the surface. Similar changes are observed in Figure 1b, which shows the FTIR spectrum of PTFE treated with PFOL and N_2_ plasma. The structure of PFOL includes a CF_3_-(CF_2_)_6_-CF_2_OH group, which is responsible for the O-H peak around 3500 cm^−1^. These findings confirm that a combination of fluorinated compound coating and a short duration of N_2_ plasma treatment for 20 s effectively modify the PTFE surface [25,26,27,28,29,30]. Longer plasma treatment times lead to weaker peaks, suggesting that the treatment removes some of the bonded molecules [4].

### 4.2. Raman Spectroscopy

**Figure 2:**
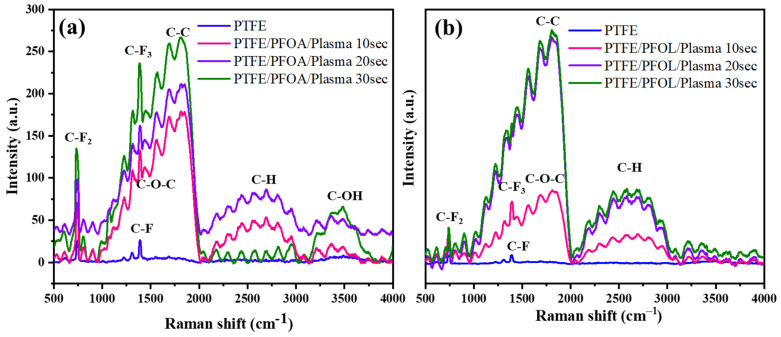
Raman spectra of (**a**) PTFE and PTFE/PFOA/N_2_ plasma and (**b**) PTFE and PTFE/PFOL/N_2_ plasma.

In the realm of vibrational spectroscopy, Raman spectroscopy was employed to scrutinize the stretching modes of PTFE and surface-modified PTFE, which are depicted in Figure 2a,b. The PTFE exhibited four distinct vibrational signatures. The asymmetric and symmetric stretches of the CF_2_ group were pinpointed at 1372 and 726 cm^−1^, respectively. The C-C bond stretching manifested as a broad band centered at 1638 cm^−1^. Additionally, a band observed at 3494 cm^−1^ was ascribed to moisture absorption, likely due to storage conditions. These prominent vibrational modes corroborate that C-F and C-C bonds are the dominant interactions within the PTFE structure.

Conversely, significant alterations in the vibrational modes were unveiled upon modification of PTFE with fluoro-compounds and N_2_ plasma treatment. For PTFE treated with PFOA/N_2_ plasma, the symmetric and asymmetric stretching modes of CF_2_ bonds were identified at 744 and 1390 cm^−1^, respectively. In the case of PTFE treated with PFOL/N_2_ plasma, these modes manifested at 746 and 1419 cm^−1^. Furthermore, new peaks emerged that correspond to side chain vibrations instigated by the PFOA coating and N_2_ plasma treatment. These peaks were observed at 1801 and 1813 cm^−1^ for C-O-C stretching, at 2644 and 2643 cm^−1^ for C-H stretching, and at 3389 and 3393 cm^−1^ for C-OH stretching [31,32,33,34,35]. These observations provide compelling evidence for extensive surface modification of PTFE attributable to the fluoro-compounds and N_2_ plasma treatment, and the identified vibrational modes align remarkably well with the stretching vibrations typically observed using FTIR spectroscopy.

### 4.3. XPS Analysis

**Figure 3:**
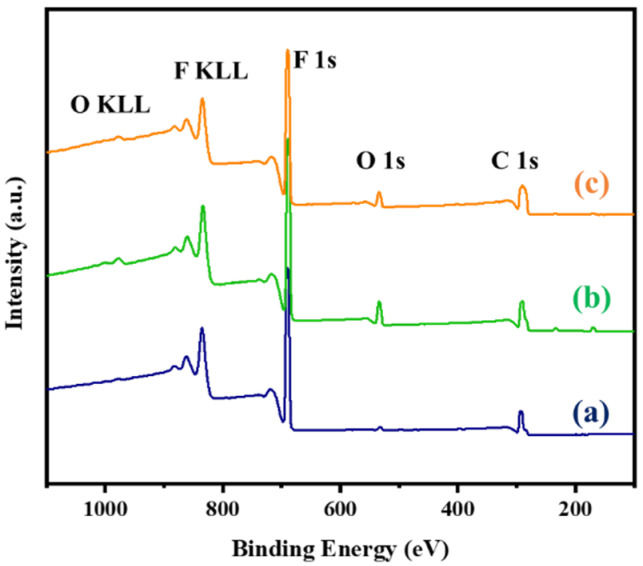
XPS survey spectra of (**a**) PTFE; (**b**) PTFE/PFOA/N_2_ plasma; and (**c**) PTFE/PFOA/N_2_ plasma.

**Figure 4:**
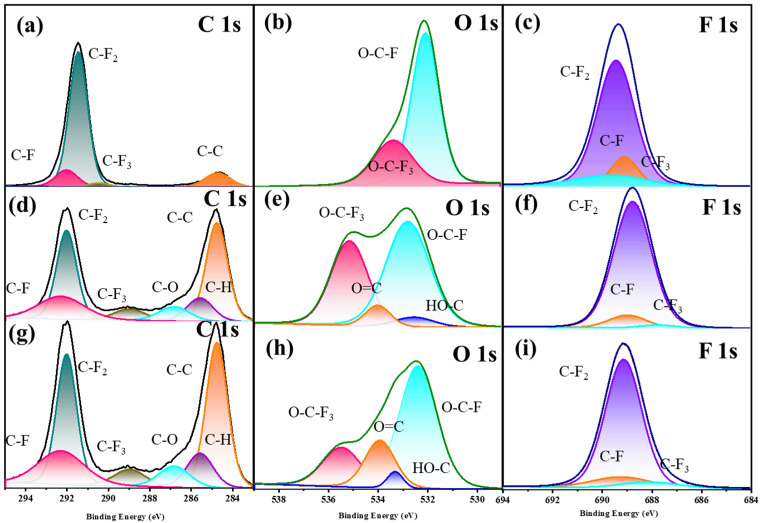
XPS deconvoluted spectra of (**a**–**c**) PTFE; (**d**–**f**) PTFE/PFOA/N_2_ plasma; and (**g**–**i**) PTFE/PFOA/N_2_ plasma.

X-ray photoelectron spectroscopy (XPS) was employed to unveil the elemental makeup and chemical state of PTFE and modified PTFE samples. Figure 3 showcases the survey spectra, revealing the characteristic peaks for carbon and fluorine in PTFE, confirming their dominance. Interestingly, the spectra for PTFE/PFOA/N_2_ plasma and PTFE/PFOL/N_2_ plasma displayed an additional peak for oxygen, signifying the inclusion of oxygen-containing functional groups due to the modifications. For a deeper understanding, deconvolution of the XPS spectra was performed and displayed in Figure 4. The deconvoluted C 1s spectrum of PTFE revealed four distinct peaks corresponding to various carbon–fluorine bonding environments: CF_2_ (292.2 eV), CF (292.6 eV), CF_3_ (291.1 eV), and C-C/surface adsorbed C-O (284.9 eV). Notably, the modified PTFE samples exhibited additional peaks between 286.2 and 284.5 eV, indicative of C-OH, C-O-C, and C-C/C-H groups, which were absent in the unmodified counterpart. The O 1s spectrum of PTFE displayed two peaks at 532.1 and 533.1 eV, attributed to surface-adsorbed oxygen atoms. However, the modified PTFE samples presented a more complex picture with additional peaks at 534.2 and 532.3 eV, corresponding to C=O/C-O and O-H groups, respectively, signifying the presence of these functional groups introduced by the modifications.

Finally, the F 1s spectrum of unmodified PTFE revealed three distinct peaks at 689.2, 689.1, and 689.8 eV, representing F_2_-C, F-C, and F_3_-C bonding environments, respectively. While the modified PTFE spectra did not exhibit new peaks, a noteworthy observation was the increased intensity of the F_2_-C peak [36,37,38,39,40]. This suggests the successful incorporation of PFOA and PFOL structures, potentially enhancing the fluorine content of the material. In conclusion, this comprehensive XPS analysis provided valuable insights into the chemical composition and surface characteristics of both PTFE and modified PTFE. This information is crucial for evaluating their performance and potential applications.

### 4.4. Apparent Contact Angle Analysis

For roughened surfaces, the wettability of the surface by the liquid (water) can be divided into four different categories, i.e., hydrophilic, hydrophobic, superhydrophilic, and superhydrophobic surfaces. The surface is said to be hydrophilic if the stable contact angle is smaller than the apparent contact angle. Whereas, in case of hydrophobic surface, the stable contact angle is larger than the apparent contact angle. A superhydrophilic surface gets completely wetted by the liquid, resulting in a contact angle of 0°, whereas a superhydrophobic surface creates a non-wettable surface with an advancing contact angle larger than 150° and contact angle hysteresis less than 10° [41,42,43,44,45].

**Figure 5:**
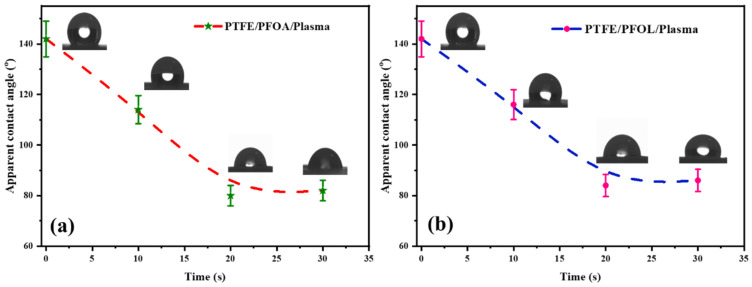
Apparent contact angle values of (**a**) PTFE and PTFE/PFOA/N_2_ plasma; (**b**) PTFE and PTFE/PFOL/N_2_ plasma.

**Figure 6:**
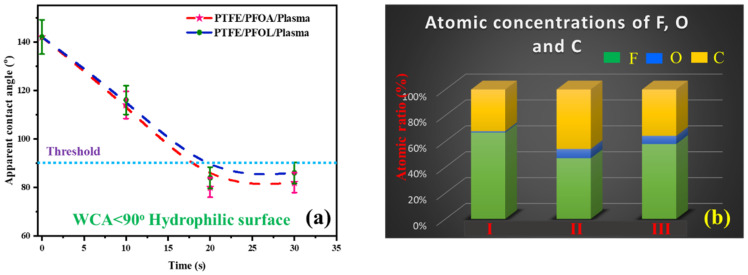
(**a**) Apparent contact angle values and (**b**) atomic concentration of (I) PTFE, (II) PTFE/PFOA/N_2_ plasma, and (III) PTFE/PFOA/N_2_ plasma.

This analysis provides valuable insights into the material’s surface behavior, as shown in Figure 5 and Figure 6. The figures not only depict the contact angle values but also include corresponding images and elemental compositions. PTFE, commonly known as Teflon^®^, is a prime example of a material with inherent hydrophobicity. As illustrated in Figure 5, PTFE exhibits a high apparent contact angle of 142°, reflecting its strong water-repelling nature. However, this property can be manipulated through various surface modification processes. Treating the PTFE surface with fluoro-compounds and N_2_ plasma treatment has been shown to significantly decrease the apparent contact angle of PTFE. PTFE surfaces treated with PFOA/N_2_ plasma for varying durations (10, 20, and 30 s) demonstrate a substantial reduction in apparent contact angles, dropping as low as 79°. Similar trends are observed with PFOL/N_2_ plasma-treated PTFE. These changes can be attributed to the introduction of hydrophilic functional groups like C-O-C and -OH onto the PTFE surface. The key takeaway is that a relatively short N_2_ plasma treatment (20 s) effectively alters the surface properties of both PFOA- and PFOL-coated PTFE, transforming them from highly hydrophobic (θ = 142°) to moderately hydrophilic (θ ≈ 80°) (comparable to He/AA plasma treatment showing 67.8° for PTFE) [18]. This shift aligns with previous research [23] suggesting that N_2_ plasma treatment can effectively render PTFE surfaces more water interactive. The underlying mechanism involves the combined effects of the fluoro-compound coatings, introducing hydrophilic groups like –COOH and –OH and the N_2_ plasma, which promotes defluorination and hydrogen absorption on the surface [7,8]. This interplay reduces the overall apparent contact angle, as confirmed by the decreased fluorine content and increased carbon and oxygen content observed in Figure 6b for modified PTFE samples.

### 4.5. SEM Analysis

**Figure 7:**
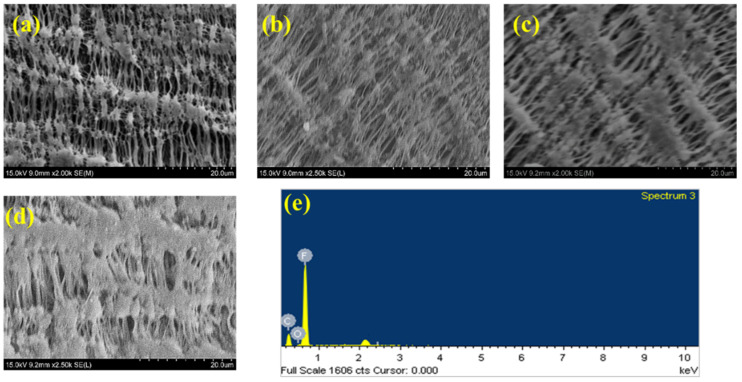
SEM images of (**a**) PTFE; (**b**–**d**) PTFE/PFOA/N_2_ plasma; and (**e**) EDX spectrum of PTFE/PFOA/N_2_ plasma.

**Figure 8:**
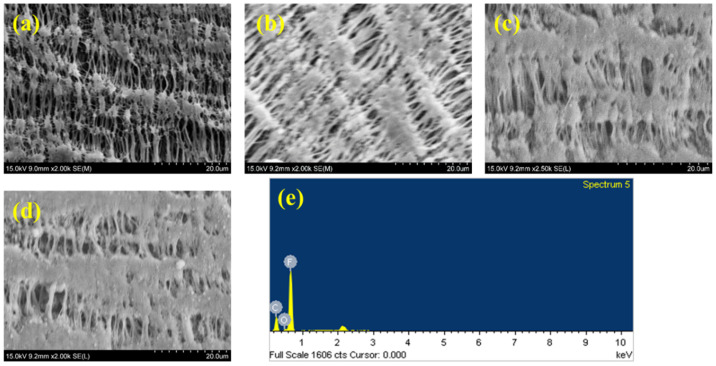
SEM images of (**a**) PTFE; (**b**–**d**) PTFE/PFOL/N_2_ plasma; and (**e**) EDX spectrum of PTFE/PFOL/N_2_ plasma.

Field-emission scanning electron microscopy (FE-SEM) was utilized to investigate the morphological features of both PTFE and modified PTFE membranes. Figure 7a presents the morphology of PTFE membrane, unveiling a network of interconnected fiber-like nodules. These fibers demonstrate a consistent alignment, with each nodule serving as a junction point for multiple fibers. This distinct reticular architecture stems directly from the melt-stretching fabrication technique employed in PTFE production. It is hypothesized that this structure, combined with the presence of -CF_2_ groups, significantly contributes to the well-established hydrophobic nature of PTFE. In stark contrast, the modified PTFE membranes (Figure 7b–d and Figure 8b–d) exhibited a drastically different surface morphology compared to their pristine counterparts. The uniform alignment of fibers was entirely disrupted, showing clear signs of tearing and breakage throughout the network. These observations strongly indicate that the combined treatment with PFOA/PFOL and plasma substantially alters the surface characteristics of PTFE. Energy-dispersive X-ray spectroscopy (EDX) analysis confirmed carbon and fluorine as the predominant elements in all samples. However, the modified PTFE membranes showed the presence of oxygen species, additionally (Figure 7e and Figure 8e) suggesting the introduction of oxygen-containing functional groups onto the modified surface of PTFE [46,47,48,49,50].

This alteration in surface morphology and chemistry could have significant implications for the membrane’s performance in various applications, particularly in terms of wettability, adhesion, and compatibility with other substances. The presence of oxygen-containing groups may enhance the membrane’s interaction with certain solvents or facilitate the attachment of functional molecules for specific applications like filtration or catalysis. Further investigation into the effects of these modifications on the membrane’s properties and performance is warranted for a comprehensive understanding and potential optimization of PTFE-based materials.

### 4.6. Stability of Surface Treatment Process

**Figure 9:**
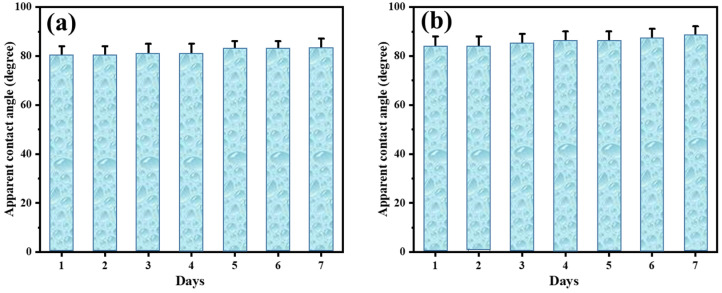
Investigating the impact of one-week aging on the apparent contact angle of (**a**) PTFE/PFOA/N_2_ plasma and (**b**) PTFE/PFOA/N_2_ plasma.

The study investigated the efficacy of two methods, PFOA and PFOL modification and N_2_ plasma treatment, in altering the surface properties of the PTFE membrane. The modified PTFE after fluoro-compound coating and N_2_ plasma treatment showed the lowest apparent contact angle, measuring at 79° (for PTFE/PFOA/N_2_ plasma) and 84° (for PTFE/PFOL/N_2_ plasma). To evaluate the durability of these modifications, apparent contact angles were measured daily for one week. The apparent contact angles increased slightly to 83° and 86° for the PTFE/PFOA/N_2_ plasma and PTFE/PFOL/N_2_ plasma-treated membranes, respectively, indicating sustained hydrophilicity (Figure 9a,b). This suggests that both methods create a long-lasting hydrophilic coating on the PTFE surface, with potential implications for various applications.

## 5. Conclusions

PTFE, known for its aversion to water (hydrophobicity), has limited its applicability in various fields. However, researchers have unlocked new potential by transforming its surface properties. This transformation is achieved through a two-pronged approach: chemical and physical modifications. The combination of chemical and physical aspects, viz., fluoro-compound coating and N_2_ plasma treatment for a short duration of 20 s, effectively alters the surface chemistry of PTFE, as confirmed by FTIR, revealing the inclusion of -COOH, C-H, and O-H groups, which is sufficient in inducing hydrophilicity. Apparent contact angle measurements showcased a dramatic shift from PTFE’s original contact angle of 142° to a significantly lower value (around 80° for both modified PTFE membranes). Beyond chemical analysis, SEM images provided visual evidence of the transformation. The initially uniform, fiber-like structure of PTFE gave way to a completely altered surface morphology after modification. These findings pave the way for precise control over PTFE’s surface properties. This combined approach opens doors to exciting possibilities: bonding PTFE with diverse materials like polymers, inorganic compounds, and even biomaterials. This expands its utility across various sectors, from medicine (where surface interactions are critical) to manufacturing (where tailored functionalities are essential). In conclusion, this research paves the way for innovative solutions by enabling the creation of PTFE with custom-designed surface properties, a development with far-reaching implications across diverse industries.

## Data Availability

The original contributions presented in the study are included in the article, further inquiries can be directed to the corresponding author.
